# INSIGHTS ON THE AGING EXPERIENCE WITHIN REMOTE CARIBBEAN ENVIRONMENTS THROUGH THE LENS OF GUANAJA, HONDURAS

**DOI:** 10.1093/geroni/igae098.0444

**Published:** 2024-12-31

**Authors:** Lee Ann Ferguson

**Affiliations:** University of Maryland Baltimore County, Baltimore, Maryland, United States

## Abstract

This qualitative study examined the aging experience of older adults residing on Bonacca Cay, a 100-acre island that serves as the central municipality of Guanaja, Honduras. Utilizing focused ethnographic methods and drawing upon the Old-Age Exclusion and Age-Space Exclusion Frameworks, the research explored socio-environmental considerations thought to influence the aging experience. The analysis of data collected through direct fieldwork, interviews, observations, and photographic journaling while on Bonacca Cay identifies eight themes elucidating the intricate interplay between sociocultural dynamics and environmental factors impacting the island’s aging population. These influential themes include (1) personal habitus of older adults, (2) vital family support and changing family structures, (3) community and social bonding, (4) cultural heritage and history, (5) built environment and service infrastructure, (6) geographic/environmental influences, (7) government, economy, and policy, and (8) access to instrumental personal care services and safety. This research also results in the recognition of both the strengths and limitations when attempting to apply either the Old-Age Exclusion or Age-Space Exclusion Frameworks to populations of older adults living on island municipalities governed by distant mainland countries. By presenting the lived realities of older adults within this remote island context, the results of this study may contribute to a more comprehensive understanding of the aging experience within low-resource Caribbean environments. It also offers valuable insights for future research and the development of tailored frameworks for studying aging in similar unique settings.

